# Venom Collection by Electrical Stimulation in the Invasive Species *Polistes dominula* Reared Using a Vespiculture Regime

**DOI:** 10.3390/molecules27248821

**Published:** 2022-12-12

**Authors:** Francesco Turillazzi, Giuseppe Pieraccini, Stefano Turillazzi, Neri Orsi Battaglini, Maurizio Severino

**Affiliations:** 1 Insect Pharma Entomotherapy S.p.A., Approved Spin Off of the University of Florence, 50100 Florence, Italy; 2 Mass Spectrometry Service Center, University of Florence, 50100 Florence, Italy; 3 Department of Biology, University of Florence, 50100 Florence, Italy; 4 Anallergo S.p.A., 50038 Scarperia e San Piero (FI), Italy

**Keywords:** *Polistes dominula*, paper wasps, electrical stimulation of venom, allergy, venom immunotherapy (VIT), vespiculture

## Abstract

Specific Venom Immunotherapy (VIT) is practiced with venom extracted from insects, and is the specific therapy used for patients highly allergic to social insect (Hymenoptera) stings. Due to the dramatic shortage of vespid species in the local environment, we coupled vespiculture techniques of *Polistes* paper wasps with a venom collection procedure based on the electrical stimulation of individuals from entire colonies. The procedure involves little to no disturbance of the individual insects, and at the same time, successfully allows for the extraction of venom containing all allergens necessary for VIT.

## 1. Introduction

Immunotherapy with social Hymenoptera venom (VIT) is mandatory for patients who suffer from severe anaphylaxis caused by the stings of these insects (Golden et al. 1980 [1]; Bilò and Bonifazi 2011 [2]; Incorvaia et al., 2011 [3]; Sturm et al., 2018 [4]; Demsar Luzar et al., 2021 [5]). The therapy consists of an induction phase followed by injections of a maintenance dose of 100 µg every 4 weeks during the first year, every 6 weeks during the second year and then every 8 weeks if venom immunotherapy is continued for more than 5 years (Bonifazi et al., 2005) [6]. It has been demonstrated that the therapy is more effective if it is performed with the venom of the hymenopteran species that is involved and is based on the results of diagnostic tests and on venom cross reactivity (Bonifazi et al., 2005) [6]. In 2019, in Italy alone, the therapy was performed almost 9000 times on species of the genus *Vespula*, 3000 times for *Vespa crabro,* and almost 6000 times for *Polistes* species (personal communication by Anallergo S.p.A.).

There exist several problems regarding the availability of venom for these therapies, especially finding the correct insects for the collection of specific venom (Feas et al., 2022) [7]. Recently, the decrease in number of various insects (including social wasps) in their distribution ranges due to habitat destruction and climate change has been reported (Forister et al., 2019 [8]; Wagner 2020 [9]) influencing the opportunity to collect vespid colonies (Hoag 2020 [10]; Aleccia 2017 [11]).

Vespiculture is an ancient technique which, in some Asian countries, is used for the rearing of wasps to obtain large quantities of insects for medicinal and culinary products. Rearing is mainly performed on hornets and species of the genus *Vespula* which can produce some of the largest colonies in social wasps. Wasps of the genus *Polistes* are seldom used due to the small size of their colonies (Van Itterbeeck et al., 2021) [12].

There exist various venom collection methods. The most commonly used (GV) is based on the dissection of the insect and the removal of its venom apparatus which includes venom glands, venom sac and sting. This is followed by the emptying of the sac either by manual squeezing or centrifugation to obtain the venom (Hoffmann et al., 2006) [13]. A technique used on honeybees is the electrical stimulation of the insects (ESV) on a grid placed at the entrance to the hive. Individual bees expel their venom which is collected on a glass plate placed under the grid (Benton et al. 1963) [14]. Importantly, ESV is also suitable for the collection of high quantities of venom from multiple individuals (Muller et al. 1981 [15]; Littler et al. 1983 [16]; Hoffmann et al. 1985 [17]; Li et al., 2013 [18]) and is made easier as honeybee colonies are maintained in manufactured apiaries. Using ESV on social wasp nests, located naturally and distributed widely in the environment, is much more problematic, however.

Attempts to collect venom of social wasps with ESV have been very limited (Eskridge et al. 1981 [19]; for a review see also Hoffmann 2006 [13]; Feas et al., 2022 [7]) and those on wasps of the genus *Polistes* are even more so (Simon and Benton 1969 [20]; Gillaspy and Grant 1979 [21]; Hoffmann et al. 1984 [22]). *Polistes* colonies are small and thus the number of individuals available for the extraction of venom is much lower when compared to the large colonies of *Vespula* or *Vespa*. *Polistes* colonies are also hard to find in large numbers.

*Polistes dominula* is a Mediterranean species which has recently invaded other countries including the USA, South Africa, Argentina, New Zealand and now also shows an extension of its range into western Asian countries. Its importance as an allergenic species therefore is now increasing rapidly, and there is a growing need for venom availability to allow for correct immunotherapy treatments in allergic patients. Historically, *P. dominula* venom collection has been performed on single insects after dissection whereafter pressing of the venom sac forces venom secretion through the sting allowing for collection with a capillary or a micropipette (Pantera et al., 2003 [23]; Bruschini et al., 2006 [24]). 

In this study we describe a novel procedure to collect venom for VIT that couples Vespiculture methods with the electrical extraction of venom of wasps belonging to colonies of *Polistes dominula* maintained in natural conditions. To validate this new technique, we performed Mass Spectrometry chemical analyses (nLC-HRMS) to confirm the presesnce of the most important allergens in the venom after its collection. 

## 2. Results and Discussion

Wasps did not appear to be severely affected by the electrical shock and we observed no wasp mortality during experiments. After 5 min of each trial the grid and glass were removed, and the wasps were allowed to pass freely. These wasps always returned to the jar after a short period. No dead wasps were observed in the base of the jar after the experiment. 

We performed chemical analyses (nLC-HRMS) of the venom collected on 26 June 2020 from 10 colonies and of the venom collected on 10 August 2020 from 17 colonies. In Table 1 and Table 2 we report the results of these analyses performed on the total of the venom collected. The tables display only the more reliable proteins and peptides detected with a Mascot score higher than 70. In both analyses we observe that all the major allergens reported for *Polistes dominula* in the literature [25] were present in detectable quantities. 

Debates on the purity of venom obtained from GV and ESV methods (both from bees and wasps) were initiated in the 1980s (Mueller et al. 1981 [15]; Littler et al. 1983 [16]; Hoffmann et al. 1985 [17]). In GV, contaminants were found to originate from venom sac substances because of the squeezing of the sac itself. In ESV experiments, instead, venom was contaminated by insect gut evacuations or other materials found on the body during reactions to the electric shock. Li et al. (2013) [18] demonstrated more definitively that honeybee venom extracted by ESV contains more venom proteins and fewer contaminant proteins than that produced by the GV extraction process. Despite this improvement, GV has remained the standard venom collection process for wasp venom. The recent study by Feas et al. (2022) [7] on *Vespa velutina* also highlighted the increased costs and times involved in GV.

The ESV method described here was applied to entire colonies of *Polistes dominula* reared in vespiaries. This differs to previous studies where wasps were mass collected in the environment and inserted into stimulation boxes (Simon and Benton 1969 [20]; Gillaspy and Grant 1979 [21]; Feas et al., 2022 [7]). In our procedure, whole colonies reared in natural conditions, were minimally disturbed and wasps were naturally motivated to defend their nests. The venom obtained from ESV contained all major allergens recently reported in the literature (including the PolD3, or Venom Dipeptidyl peptidase) [25].

As analyses were directed to detect the heaviest component of the venom, where the major allergens are reported, more volatile substances which can have important roles in the biology of wasp colonies [24] were probably lost but antimicrobial peptides such as Dominulins (Turillazzi et al., 2006) [26] were clearly present in the collected matter. 

## 3. Materials and Methods

### 3.1. Vespiculture

**Figure 1:**
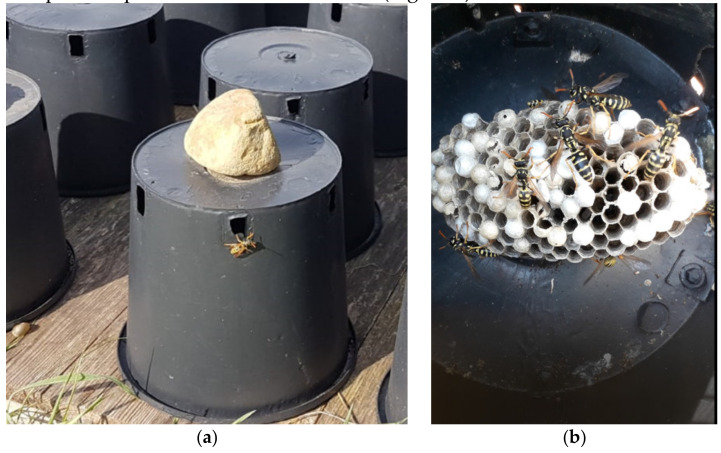
(**a**) Outside and (**b**) inside of plastic jars used to rear *Polistes dominula* colonies.

*Polistes dominula* is a common wasp in Italy with colonies rarely reaching 100 adult individuals. Nests are usually founded in closed, warm and ventilated places making this species suitable for rearing under controlled conditions in suitable containers. Colonies are collected in the field and transferred to plastic containers with slots for ventilation and exit and entry by workers. Wasps used the containers to establish new colonies even in the following year. We organized “vespiaries” in different places (4 sites) around Florence using plastic plant nursery jars of 16 × 20 cm with six slots 2 × 1 cm as containers. The jars were placed upside down on flat surfaces (Figure 1).

### 3.2. Collection of Venom

**Figure 2:**
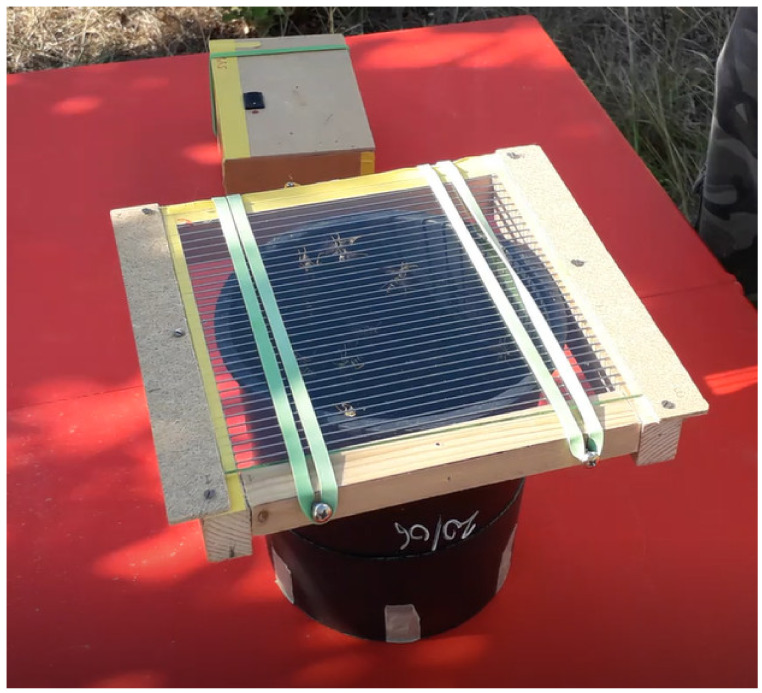
Apparatus to collect venom from wasps belonging to a single colony.

Well-established colonies with populations varying from 20 to 50 adult individuals were used for the collection of venom. During collection, after sealing the slots in each of the jars, we moved the jar gently onto a grid (20 × 20 cm) made of steel filaments (1 mm in diameter) mounted 0.5 cm apart on a wooden frame, with a clean glass plate (20 × 20 cm) located underneath. We then turned the jar and plate over and exposed the grid + glass to sun light (Figure 2).

**Figure 3:**
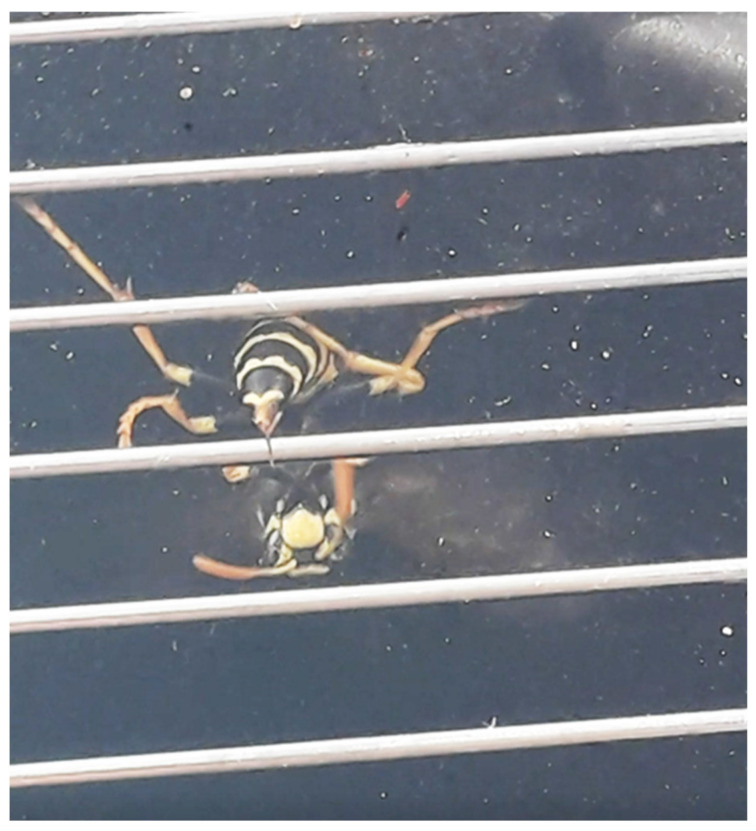
A wasp stinging the glass after electrical stimulation.

Wasps flew towards the light and encountered the grid which was connected to an electric pulse device. constructed with a kit supplied by New Hobby Ltd. (Sofia, Bulgaria; newhobby.world@gmail.com (accessed on 1 May 2020)) and designed for the collection of honeybee venom. (https://www.facebook.com/newhobby.eu/posts/new-version-of-bee-venom-collector-controller-ver-pro-is-ready-now-are-added-opt/2202935649962432/ (accessed on 1 May 2020)). Output voltage was kept around 10 volts. Wasps stung and left their venom on the glass (Figure 3).

After several preliminary trials performed on colonies reared in the laboratory, we performed the experimental electrical stimulation over a period of 58 days, from 15 June to 11 August 2020 (when the annual cycle presents a maximum of colonial population), on a total of 144 different colonies, reared in 4 different vespiaries. Each colony had approximately 15 adult wasps. Fifteen of these colonies were re-extracted after 3 days. All the trials were performed on sunny days, with ambient temperatures over 20 °C in order to work with more active individuals 

After stimulation of a variable number of colonies, any venom collection glass was removed, transferred to the laboratory and stored at −20 °C until venom recovery and analysis. In contrast to the venom of *Apis, Bombus* and *Vespa velutina*, *Polistes* venom remains sticky and water soluble. Consequently, we poured 300 mL of distilled water over the glass after which we scraped the diluted venom with a razor (avoiding any faeces that had been deposited) and collected the solution with a micropipette. This was ultimately transferred to 0.5 mL vials.

### 3.3. Chemical Analyses

After centrifugation (10,000× *g* rpm for 15 min at 4 °C), the total protein concentration in the venom solution was estimated using the assay kit and a Qubit fluorometer (Thermo Fisher Scientific, Milan, Italy). The analysis of the samples was performed as described in Dani and Pieraccini 2020 [27] with minor modifications. In brief, the volume corresponding to 10 µg of proteins was taken from each sample. After reduction and alkylation, a LysC and trypsin digestion was performed, and the peptides purified and enriched by stage tip. The samples were taken to a final volume of 20 µL with 0.5% acetic acid and injected (1 µL volume) into the nLC-nESI HRMS/MS instrument (Thermo Fisher Scientific, Bremen, Germany) operating in gradient mode and acquiring data in Data Dependent Acquisition (DDA), as described in [27]. The column was an Acclaim^®^ PepMap 100 C18 (3 µm, 75 µm × 150 mm) and the flow rate 300 nL/min (Thermo Fisher Scientific, Milan, Italy). HRMS spectra were recorded at 60,000 resolution (at 400 *m*/*z*) in the range 350–2000 *m*/*z*. Quadrupole ion trap MS/MS spectra were recorded for the six most intense peaks in the HRMS scan.

Mascot 2.4 (Matrix Science Ltd., London, UK) was used to analyze the acquired data. The generated tryptic peptides were searched against a database containing the *Polistes* proteins (created on 16 July 2020), including modifications as cysteine carbamidomethylation and methionine oxidation and considering a maximum of 3 missed cleavages. A 1% false discovery rate (FDR) was imposed and protein identification accepted for a Mascot probabilistic score higher than 70. The results were then filtered to highlight proteins that are well documented to be allergens in wasp venom [25]. 

## 4. Conclusions

The electrical collection of venom from wasps reared under a vespiculture regime is an efficacious procedure for the collection of venom for immunotherapy (VIT). Importantly, this procedure does not require the sacrifice of wasp colonies and thus avoids negative impacts on the population of important species in the local ecosystem (Bork et al., 2021) [28]. We do not yet know if this method results in a lower or higher production of secretion with respect to GV procedures and, certainly, with this technique more volatile components of the venom, which could be important in the wasp biology are lost. Further research is needed to focus on improving of the quality of vespiculture farming, wasp population management as wells as types of stimulation devices.

## Figures and Tables

**Table 1:** Chemical analyses of the total venom collected on 26 June 2020 from 10 colonies of *P. dominula* with electrical stimulation. The table presents only the more reliable proteins, known allergens, detected with a Mascot score higher than 70.

Accession	Score	Mass	Num. of Significant Matches	Num. of Significant Sequences	emPAI	Description
P83377	**2297**	23138	138	9	4.82	**Venom allergen 5** OS = *Polistes gallicus* OX = 34730 PE = 1 SV = 1
Q7Z269	**1952**	30784	69	10	4.17	**Venom serine protease** OS = *Polistes dominula* OX = 743375 PE = 2 SV = 1
Q9U6V9	**1767**	42992	105	9	1.26	**Hyaluronidase** (Fragment) OS = *Polistes annularis* OX = 27505 PE = 1 SV = 1
Q6Q251	**1706**	35037	84	18	6.29	**Phospholipase A1 2** (Fragment) OS=*Polistes dominula* OX = 743375 PE = 2 SV = 1
Q6Q250	**1185**	34997	73	18	5.7	**Phospholipase A1 3** (Fragment) OS = *Polistes dominula* OX = 743375 PE = 2 SV = 1
Q9U6W0	**45**	33462	6	3	0.33	**Phospholipase A1** OS = *Polistes annularis* OX = 27505 PE = 2 SV = 1
P0C1M6	**142**	1855	9	2	9.28	**Dominulin-A** OS = *Polistes dominula* OX = 743375 PE = 1 SV = 1
B1A4F7	**107**	88868	2	1	0.04	**Venom dipeptidyl peptidase 4** OS = *Vespula vulgaris* OX = 7454 PE = 1 SV = 1
P0C1M7	**94**	1910	8	2	9.28	**Dominulin-B** OS = *Polistes dominula* OX = 743375 PE = 1 SV = 1
P85873	**83**	1358	13	2	83.54	**Wasp kinin PMM1** OS = *Polistes major* OX = 91420 PE = 1 SV = 1

**Table 2:** Chemical analyses of the total venom collected on 10 August 2020 from 17 colonies of *P. dominula* with electrical stimulation. The table displays only the more reliable proteins, and known allergens, detected with a Mascot score higher than 70.

Accession	Score	Mass	Num. of Significant Matches	Num. of Significant Sequences	emPAI	Description
Q6Q249	**2073**	35001	95	17	9.55	**Phospholipase A1 4** (Fragment) OS = *Polistes dominula* OX = 743375 PE = 2 SV = 1
Q6Q252	**1768**	37535	82	16	7.29	**Phospholipase A1 1** OS=*Polistes dominula* OX = 743375 PE = 1 SV = 1
P81656	**1426**	25430	64	10	7.16	**Venom allergen 5** OS = *Polistes dominula* OX = 743375 PE = 1 SV = 2
Q7Z269	**1070**	30784	36	8	3.21	**Venom serine protease** OS = *Polistes dominula* OX = 743375 PE = 2 SV = 1
Q9U6V9	**607**	42992	23	5	0.56	**Hyaluronidase** (Fragment) OS = *Polistes annularis* OX = 27505 PE = 1 SV = 1
P0C1M6	**139**	1855	7	1	2.21	**Dominulin-A** OS = *Polistes dominula* OX = 743375 PE = 1 SV = 1
P85873	**111**	1358	19	2	83.54	**Wasp kinin PMM1** OS = *Polistes major* OX = 91420 PE = 1 SV = 1
B1A4F7	**93**	88868	2	1	0.04	**Venom dipeptidyl peptidase 4** OS = *Vespula vulgaris* OX = 7454 PE = 1 SV = 1
P0C1M7	**82**	1910	4	1	2.21	**Dominulin-B** OS = *Polistes dominula* OX = 743375 PE = 1 SV = 1

## Data Availability

Not applicable.
